# Thermo-Responsive Cellulose-Based Material with Switchable Wettability for Controllable Oil/Water Separation

**DOI:** 10.3390/polym10060592

**Published:** 2018-05-28

**Authors:** Wenbo Chen, Hui He, Hongxiang Zhu, Meixiao Cheng, Yunhua Li, Shuangfei Wang

**Affiliations:** 1 College of Light Industry and Food Engineering, Guangxi University, Nanning 530004, China; ilaureshang@163.com (W.C.); cmx29192@163.com (M.C.); liyunhua199202@163.com (Y.L.); wangsf@gxu.edu.cn (S.W.); 2 Guangxi Key Laboratory of Clean Pulp & Papermaking and Pollution Control, Nanning 530004, China

**Keywords:** thermo-responsive, controllable oil/water separation, in situ variable-temperature NMR, cellulose, chemical grafting

## Abstract

A thermo-responsive cellulose-based material (cellulose-*g*-PNIPAAm) was prepared by grafting *N*-isopropylacrylamide (NIPAAm) onto bagasse pulp cellulose via Ce (IV)-initiated free radical polymerization. The surfaces of the obtained cellulose-*g*-PNIPAAm paper showed a rapid wettability conversion from being hydrophilic (water contact angles (WCA) of 0°) at 25 °C to becoming hydrophobic (WCA of 134.2°) at 45 °C. Furthermore, the thermo-responsive mechanism of cellulose-*g*-PNIPAAm was examined by the in situ variable-temperature ^13^C NMR, ^1^H NMR and AFM analysis. At the same time, the resulting cellulose paper was applied for a switchable separation of oil/water mixtures. Water can pass through the paper under 45 °C, while oil is kept on the paper. When the temperature is above 45 °C, oil can permeate through the paper, while water cannot pass through the water. Moreover, the paper exhibited excellent regeneration performance after five cycles and maintained its switchable wettability.

## 1. Introduction

Wastewater containing industrial oil and marine oil spill accidents pose threats to humans and the environment [1,2,3]. Effectively separating the oil from the wastewater has been a global challenge. Interface science, especially with respect to the special surface wettability, has focused on finding a healthy solution to this environmental issue [4]. A number of super-wetting materials have been applied for oily wastewater separation through the manipulation of both the surface structure and chemical composition [5,6,7,8,9]. According to the wettability differences, there are three types of separation materials: “oil-removing” type materials [10,11] with super-hydrophobicity and super-oleophilicity; “water-removing” type materials [12,13] with super-hydrophilicity and super-oleophobicity; and smart controllable separation materials [14]. Compared with the other two types of materials, the smart controllable separation materials have more advantages in simplifying separation devices, accelerating the separation rate, increasing the stability, reducing the energy use and providing promising potential in fabricating intelligent controllable materials [15,16]. When the external stimuli change, such as temperature [17,18], magnetism [19], light [20], pH [21], ion strength [22], enzyme [23] and antigen [24], such smart materials are able to achieve the switch of surface wettability. As for a thermo-responsive material, a considerable number of compounds can be used to realize its responsibility, such as *N*-isopropylacrylamide (NIPAAm) [25], *N*,*N*-diethylacrylamide (DEAAm) [26], *N*-vinylcaprolactam (VCL) [27], copolymers of P(MEO2MA-*co*-OEGMA)]-*b*-PCL-*b*-P(MEO2MA-*co*-OEGMA) [28] and vinyl alcohol [29]. Poly(NIPAAm) and its derivatives have attracted great interest due to the thermo-responsive hydrophilic–hydrophobic phase transition of the PNIPAAm molecules [30], which have wide applications in various fields, such as biomedical areas [31,32,33] and oil/water separation [34,35]. Zhou et al. [36] create thermo-responsive stainless steel meshes for tunable oil/water separation by grafting a well-defined block thermo-responsive copolymer via sequential photo ATRP. In this study, the contact angle of the membrane could be changed from 0° at 25 °C to 134.2° at 50 °C. Song et al. [37] fabricated a poly (*N*-isopropylacrylamide) (PNIPAAm)-modified rough copper mesh film, which shows good water permeability (CA ≈ 34.6 ± 6.1°) at low temperatures (below 25 °C). When the temperature is above 40 °C, the film is impermeable to water (CA ≈ 156.5 ± 5.1°). This phenomenon illustrates that the film can be used effectively in controllable filtration and separation as well as other relevant applications. However, the majority of substrates of oil/water separation materials are metal meshes, which are difficult to degrade and may further result in secondary pollution to the environment. Furthermore, the thermo-responsive groups and copolymers are physically loaded onto the metal meshes, which affect the regeneration performance of the materials. Thus, it is necessary to choose another substrate instead of the mesh substrates to develop an environmentally friendly material.

Natural cellulose is one of the largest renewable resources and it has been considered to be a substrate of biomass-based flexible material as it is inexpensive, biocompatible, environmentally adaptable and easily modified [38,39]. Thus, the thermo-responsive groups and copolymers can be grafted onto the surfaces of cellulose to prepare the thermo-responsive material for controllable oil/water separation.

As previously reported, many researchers have synthesized temperature-sensitive cellulose-based materials by using commercial pure cellulose via ATRP or RAFT [40,41,42], which has a relatively low grafting degree of PNIPAAm. As a sequel to the present study, bagasse cellulose, a largest biomass resource, was used as a substrate to prepare high-value biomass products. Bleached bagasse pulp cellulose is not pure cellulose as it is instead a material with a cellulose content of about 70–90%. However, to our knowledge, a relatively simple, efficient and environmental method for the preparation of higher-grafting initial efficiency has not reported in the literature, such as the ^60^Co γ-ray irradiation technique used to pre-treat the bleached bagasse pulp cellulose. Therefore, it is of great significance to conduct some research on developing low-cost and environment-friendly smart materials with thermally switchable hydrophobicity functions, which could greatly widen the applications of the materials being constructed.

In this study, a thermo-responsive cellulose-based material (Cellulose-*g*-PNIPAAm) was prepared by grafting NIPAAm onto bagasse pulp cellulose via Ce (IV)-initiated free radical polymerization. The different thermo-responsiveness of the cellulose-*g*-PNIPAAm can be tailored through controlling the grafting degree of NIPAAm onto bagasse pulp cellulose. The chemical construction, thermo-responsive performance, oil/water separation, long-term regeneration stabilities and temperature-responsive mechanism of the resulting cellulose-based material were also studied.

## 2. Materials and Methods

### 2.1. Materials and Reagents

The bleached bagasse cellulose was obtained from the Guitang paper industry of Guangxi, (Guitang, China). Ce (IV) ammonium nitrate (CAN), sodium hydroxide (NaOH), acetone, ethanol, hydroquinone, methylene blue and sudan (III) were purchased from the Aladdin Corporation (Shanghai, China). *N*-isopropylacryalamide (NIPAAm) was purchased from the Aladdin Corporation (Shanghai, China), which was used after being purified by a recrystallization method from hexane and dried at 25 °C in a vacuum desiccator.

### 2.2. Preparation of Thermo-Responsive Cellulose-Based Material

**Figure 1:**
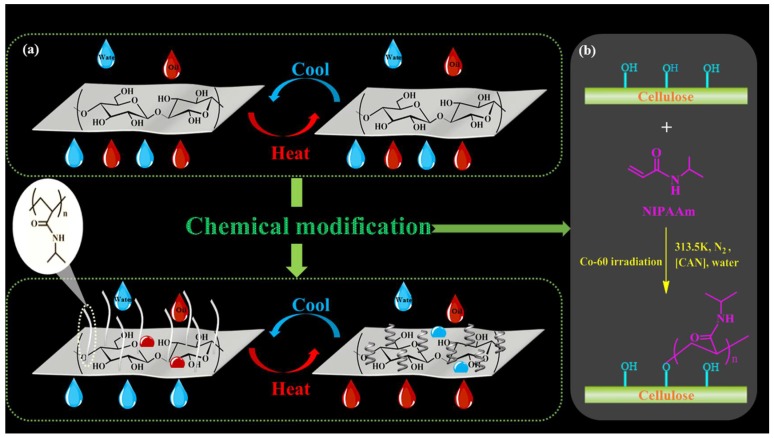
The fabrication of the cellulose-*g*-PNIPAAm paper through chemical modification (**a**); the schematic description of the preparation of cellulose-*g*-PNIPAAm (**b**).

Cellulose-*g*-PNIPAAm was prepared via grafting PNIPAAm onto the surface of bagasse cellulose in a deionized water solvent system through the “one-step” method, which is shown in Figure 1b. In a typical synthesis procedure, a certain amount of bleached bagasse pulp with a cellulose content of 73% was first weighed after being impregnated in the 5% sodium hydroxide solution at 25 °C for about 3 h. After this, it was washed several times with deionized water and ethanol until the filter liquor became neutral, before it was placed in a 105 °C oven and dried for 4 h to obtain the pre-treated bleached bagasse pulp cellulose. After this, the cellulose was subjected to ^60^Co γ-ray irradiation at a dosage rate of 0.837 kGy/h to obtain pre-irradiated cellulose. The monomer (NIPAAm) was grafted onto the pre-irradiated cellulose by the following procedure: 2.0 g of the pre-irradiated cellulose was added to a three-necked flask containing 150 mL of a solution of CAN at 40 °C under N_2_ protection. NIPAAm was subsequently added to the above solution, which was maintained at 40 °C for 12 h in order to carry out the graft copolymerization reaction between cellulose and NIPAAm. The reaction was arrested by adding a 1.0% solution of hydroquinone at a fixed time of the reaction. In order to remove the unreacted monomer (NIPAAm) and its low molecular weight homopolymer (PNIPAAm), the resulting reaction product was washed several times with cold and hot deionized water, before these byproducts were extracted by acetone and 95% ethanol, respectively. Finally, the product was dried under a vacuum to a constant weight at 60 °C for 12 h [43]. A total of 1.88 g of dried product (quantitatively 60 ± 3 g/m^2^) was poured into a disperser equipped with 4 L of distilled water for about 30 min, before the disperse uniform pulp was poured into a paper shaper to be molded into a wet paper. Vacuum drying apparatuses were used to dry the paper for 5–7 min at 96 KPa and a dry paper material was obtained. The obtained thermo-responsive cellulose-based material was labeled as cellulose-*g*-PNIPAAm.

The grafting degree of cellulose-*g*-PNIPAAm (*G*, %) was calculated from elemental analysis by the following equation (Equation (1)):

(1) $$G=\left(\frac{\frac{N\%\times \left(M-1\right)}{14}}{1-\frac{N\%\times \left(M-1\right)}{14}}\right)\times 100\%,$$

where *M* is the molecular weight of monomer NIPAAm (113.16).

### 2.3. Oil/Water Adsorption and Desorption Experiment

The as-prepared paper products prepared from cellulose and cellulose-*g*-PNIPAAm were immersed into an oil/water mixture for the adsorption experiment. In an oil-in-water mixture, the oil was stained by sudan (III). In a water-in-oil mixture, the water was stained with methylene blue. The temperature of the oil/water mixture was increased by heating from 25 °C to 50 °C. The oil desorption experiment was conducted by putting the oil-containing cellulose-*g*-PNIPAAm paper into a water solution with a temperature of 25 °C (<Lower Critical Solution Temperature) for 24 h.

### 2.4. Oil/Water Separation Experiment

The oil/water separation experiments were performed through a separation device for the as-prepared cellulose paper and cellulose-*g*-PNIPAAm paper under the action of gravity separation. Typically, the separation device, which consists of a customized sand-core glass joint coupled with two tubes on both sides, holds the paper between the two tubes firmly. The diameter of the sand-core glass joint was 40 mm. The oil/water mixtures (30 *v*/*v* %) were poured onto the as-prepared paper at 25 °C and the water was collected in the beaker. Oil was successively poured onto the paper. At the same time, the paper was in situ heated to 50 °C with an 800 W hair dryer for 2.5 min. The oil was collected in the beaker, while the water was poured onto the paper.

To measure the flux of the water, 50 mL of oil/water mixture were poured into the separation device. The separation time was recorded and the separation flux of the cellulose-*g*-PNIPAAm paper was calculated according to Equation (2):

(2) $$\mathrm{flux}=\frac{V}{St},\;$$

where *V* is the volume of the permeate water, *S* is the valid area of the paper and *t* is the testing time. The separation flux was obtained from the average values of the five measurements.

The oil was dyed red by sudan (III). Both the cellulose paper and the as-prepared cellulose-*g*-PNIPAAm paper were tested under two different temperature conditions. The absorbed oil in the inner paper could be consecutively removed by adjusting the temperature and thus, the materials could be reused.

### 2.5. Physical and Chemical Characterization

Fourier Transform Infrared Spectroscopy (FTIR) (Bruker T27, Karlsruhe, Germany), elemental analysis (EA) (Elementar Vario EL, Hanau, Germany), field emission scanning electron microscopy (FE-SEM) (Hitachi SU8220, Tokyo, Japan), energy dispersive spectroscopy (EDS), in situ variable-temperature solid-state CP/MAS ^13^C Nuclear Magnetic Resonance spectroscopy (NMR) (AVANCE AV400, Karlsruhe, Germany), in situ variable-temperature MAS ^1^H Nuclear Magnetic Resonance spectroscopy (NMR) (Agilent 600DD2, CA, USA) and X-ray photoelectron spectroscopy (XPS) (Thermo-VG Scientific ESCALAB 250, Waltham, MA, USA) were employed to determine the composition, chemical structure and thermo-responsive mechanism of the samples. Furthermore, the thermal stability of the samples was determined by thermo-gravimetric analysis (TGA) (NETZSCH STA499F5, Serb, Germany), which was performed in the range of 100–700 °C with a heating rate of 10 °C/min under nitrogen. The wettability of the materials was determined by Contact angles (CA) (DSA100, Hamburg, Germany). The surface roughness of the materials was determined via atomic force microscopy (AFM) (Hitachi 5100N, Tokyo, Japan).

## 3. Results and Discussion

### 3.1. Chemical Structure and Thermal Stability of Cellulose-g-PNIPAAm

**Figure 2:**
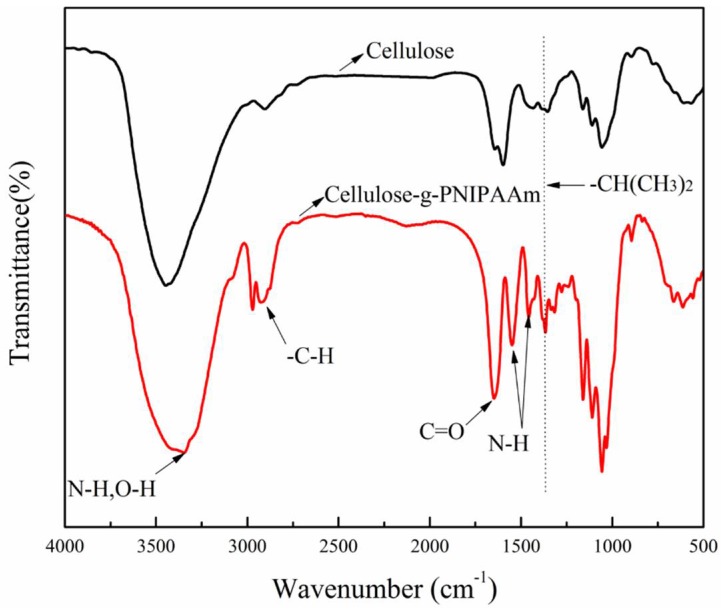
The Fourier Transform Infrared spectroscopy spectra of the cellulose and cellulose-*g*-PNIPAAm.

The FTIR spectra of the cellulose and cellulose-*g*-PNIPAAm are shown in Figure 2. It can be seen that the FTIR spectrum of cellulose shows peaks at 3450 cm^−1^ (–O–H stretching), 2914 cm^−1^ (–C–H stretching), 1057 cm^−1^ (–C–O–C pyranose ring skeletal vibration) and 897 cm^−1^ (β-glycosidic linkages). In contrast, the spectrum of cellulose-*g*-PNIPAAm shows new characteristic adsorption peaks at 1540 cm^−1^, (N–H stretch vibration of PNIPAAm), 1650 cm^−1^ (C=O stretching vibration of amide groups), 1456 cm^−1^ (asymmetric bending vibration of –CH_2_– groups) and 1364 cm^−1^ (isopropyl (–CH(CH_3_)_2_) groups) [44]. The combination of these four new characteristic adsorption peaks indicates that NIPAAm was successfully grafted onto the cellulose via Ce (IV)-initiated free radical polymerization.

**Figure 3:**
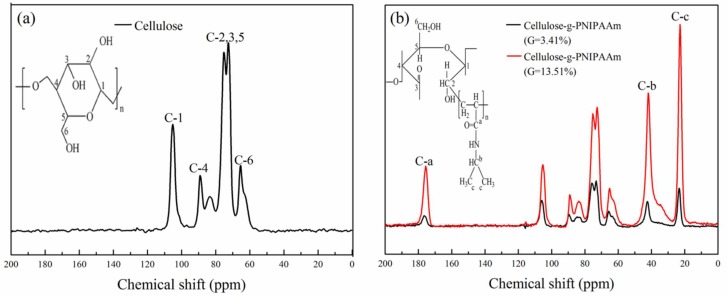
The ^13^C solid-state Nuclear Magnetic Resonance spectra of cellulose (**a**) and cellulose-*g*-PNIPAAm (G_EA_ = 3.14% and 13.51%) (**b**).

In addition to the FTIR spectra, cellulose and cellulose-*g*-PNIPAAm were also investigated by ^13^C solid-state NMR spectra (Figure 3). Compared with the ^13^C NMR spectrum of the cellulose, the spectrum of cellulose-*g*-PNIPAAm showed new characteristic peaks at 175.5 ppm (C-a, corresponds to the amide carbonyl C atom), 41.9 ppm (C-b, corresponds to the sp3-hybridized C atom in the poly (*N*-isopropylacrylamide) backbone (C–N)) and 22.6 ppm (C-c, corresponds to sp3-hybridized C atom in the poly (*N*-isopropylacrylamide) backbone (–CH–(CH_3_)_2_). These peaks clearly indicate the presence of poly (*N*-isopropylacrylamide) in cellulose-*g*-PNIPAAm. Furthermore, the intensities of the characteristic peaks increased with an increase in the grafting degree of cellulose-*g*-PNIPAAm. In addition, no substantial modifications were observed for the cellulose backbone in the 50–110 ppm region, indicating that the graft copolymerization reaction did not remarkably affect the backbone structure of cellulose.

**Figure 4:**
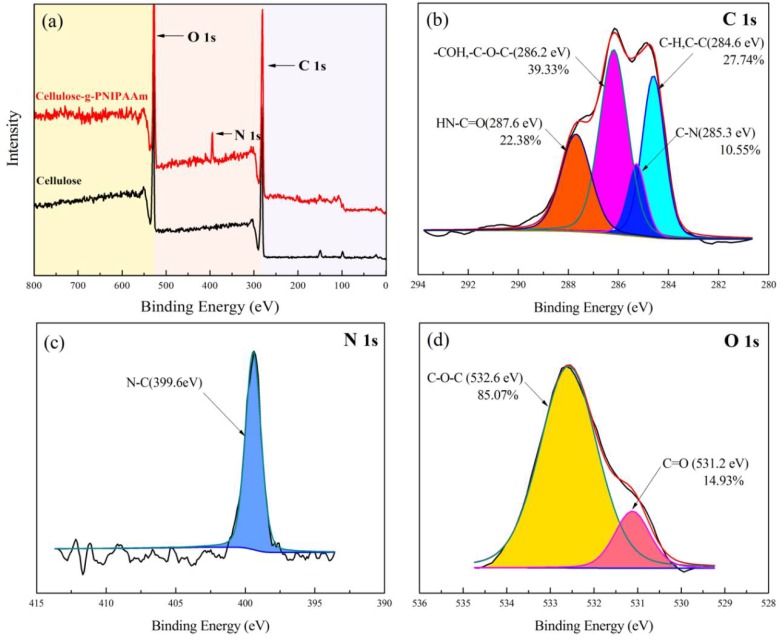
The wide-scan X-ray Photoelectron Spectroscopy spectra of cellulose and cellulose-*g*-PNIPAAm (G_EA_ = 42.28%) (**a**); the deconvolved curves of the C 1s spectrum (**b**); the deconvolved curves of the N 1s spectrum (**c**); and the deconvolved curves of the O 1s spectrum (**d**) of cellulose-*g*-PNIPAAm.

As shown in the XPS results (Figure 4a), no nitrogen was detected on the surface of the cellulose, while the nitrogen content was 5.50% on the surface (10 nm) of cellulose-*g*-PNIPAAm. In the C 1s spectrum of cellulose-*g*-PNIPAAm (Figure 4b), there were four peaks at 284.6 eV, 285.3 eV, 286.2 eV and 287.6 eV, which were attributed to the C–H/C–C, C–N, C–O–C/C–O-H and H–N–C=O groups, respectively. The H–N–C=O group content on the cellulose-*g*-PNIPAAm surface was calculated to be 22.38%. The nitrogen-containing groups were further resolved into secondary amino groups (C–NH–) of NIPAAm by the N 1s peak processing of the XPS spectra (Figure 4c). More specifically, the two deconvolved peaks of O 1s in cellulose-*g*-PNIPAAm (Figure 4d) were 532.6 eV and 531.2 eV, respectively, which correspond to the C–O–C in the backbone of the cellulose and the C=O in NIPAAm. The C=O group content on the surface of cellulose-*g*-PNIPAAm was 14.93%. The combination of the C 1s, O 1s and N 1s spectra of cellulose-*g*-PNIPAAm demonstrates that a large number of *N*-isopropylacrylamide groups (H–N–C=O groups in the C 1s spectrum, C=O groups in the O 1s spectrum and C–NH– groups in the N 1s spectrum) was introduced to the surface of cellulose.

**Figure 5:**
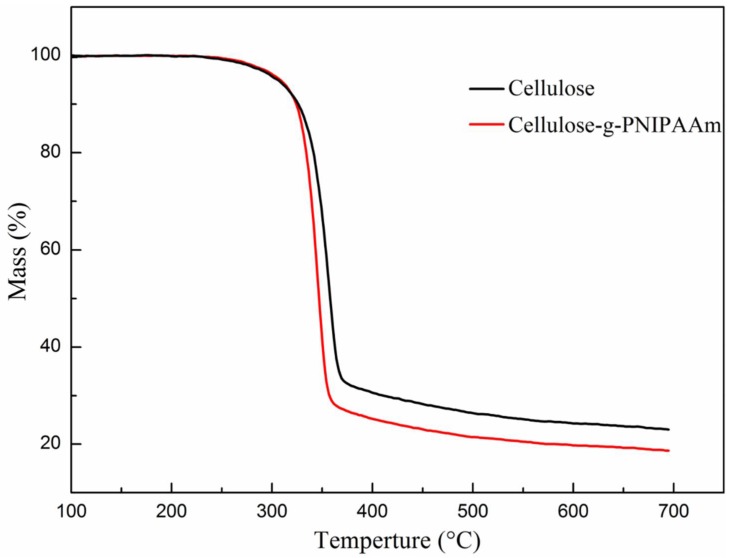
The thermal stability of the cellulose and cellulose-*g*-PNIPAAm.

In order to detect the thermostability of the cellulose before and after modification, thermogravimetric analyses were conducted on cellulose and cellulose-*g*-PNIPAAm (Figure 5). The weight of cellulose and cellulose-*g*-PNIPAAm did not begin to reduce until the temperature was raised to 260 °C. The undecomposed component of cellulose and cellulose-*g*-PNIPAAm at 700 °C was ash, indicating that cellulose and cellulose-*g*-PNIPAAm could be practically used at temperatures under 260 °C.

### 3.2. Thermo-Responsive Mechanism of Cellulose-g-PNIPAAm

**Figure 6:**
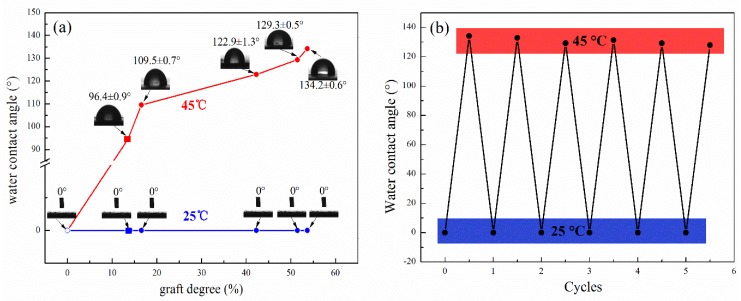
The variation of the Water Contact Angle test of cellulose and cellulose-*g*-PNIPAAm with different graft degrees at 25 °C and 45 °C, respectively (**a**); Reversible WCA transition of the product at 25 °C and 45 °C, respectively (**b**).

As shown in Figure 6, the surfaces of the paper made by cellulose-*g*-PNIPAAm with different grafting degrees showed a rapid wettability conversion from being hydrophilic (WCA = 0°) at 25 °C to becoming hydrophobic (WCA > 90°) at 45 °C. The WCA of the cellulose-*g*-PNIPAAm paper increased with a higher grafting degree. The WCA reached the maximum value (134.2°) when the grafting degree of cellulose-*g*-NIPAAM was 53.68% (Figure 6a). Meanwhile, the cellulose-*g*-PNIPAAm was processed for five cycles of hydrophilicity (at 25 °C) and hydrophobicity (at 45 °C) switching, with the results shown in Figure 6b. After five cycles, no significant changes in the WCA at 25 °C and 45 °C were observed, while the WCA of the regenerated cellulose-*g*-PNIPAAm remained at 0° (at 25 °C) and 134.2 ± 0.6° (at 45 °C), respectively. It was obvious that the thermo-responsive cellulose-based material could remain stable after multiple regeneration cycles and maintain its switchable wettability.

The as-prepared cellulose-*g*-PNIPAAm showed a highly switchable wettability from being hydrophilic to becoming hydrophobic in response to a change in the temperature. The thermo-responsive mechanism of cellulose-*g*-PNIPAAm could be revealed as follows: the competition of inter-molecular/intra-molecular H-bonding between the C=O and N–H groups in the PNIPAAm chains in response to temperature leads to a conformation change of the PNIPAAm chains within a narrow range. At 25 °C, the loosely coiled conformation of the PNIPAAm chains and intermolecular H-bonding with water molecules leads to the hydrophilic surface of the cellulose-*g*-PNIPAAm paper. However, the intramolecular hydrogen bonding between the C=O and N–H groups in the PNIPAAm chains results in a compact and collapsed conformation of the PNIPAAm chains at 45 °C, which makes it difficult for the hydrophilic C=O and N–H groups to interact with the water molecules. Therefore, the surface of the cellulose-*g*-PNIPAAm paper converted from being hydrophilic to becoming hydrophobic.

**Figure 7:**
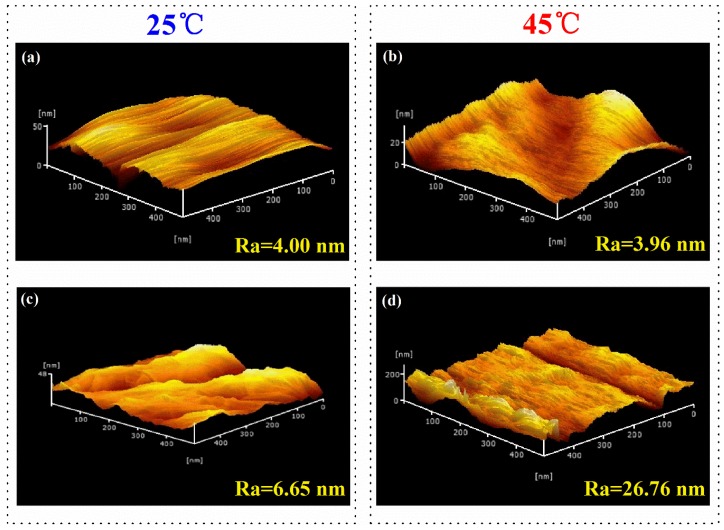
The Atomic Force Microscope spectra of cellulose at 25 °C (**a**) and 45 °C (**b**); and cellulose-*g*-PNIPAAm at 25 °C (**c**) and 45 °C (**d**).

To further find out the effect of the roughness on the surface wettability, AFM was used to probe the surface roughness of the cellulose and cellulose-*g*-PNIPAAm at different temperatures in the 500-nm data scale, with the 3D images shown in Figure 7. Based on the AFM image, the arithmetic average roughness (Ra) of the cellulose paper was 4.00 nm at 25 °C (Figure 7a), which was similar to that (3.96 nm) at 45 °C (Figure 7b). This phenomenon indicates that the nanoscale roughness of the surface of the cellulose paper at low and high temperatures did not significantly change, while the Ra values of the cellulose-*g*-PNIPAAm paper were 6.65 nm at 25 °C (Figure 7c) and 26.76 nm at 45 °C (Figure 7d). Compared with cellulose paper, the nanoscale roughness of the surface of cellulose-*g*-PNIPAAm paper at a low temperature (25 °C) increased, while the value at a high temperature (45 °C) increased greatly, indicating that the water is a good solvent as the PNIPAAm chain exist as coils below the temperatures of 45 °C. At temperatures above 45 °C, the hydrogen bonding of the water with the amide groups is disrupted. Subequently, the attractive inter-segment interactions between the isopropyl groups dominate. Therefore, the PNIPAAm chain collapse to globules [45].

**Figure 8:**
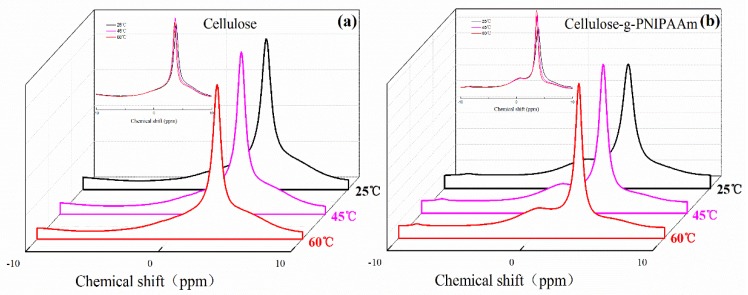
The in situ variable-temperature ^1^H solid-state Nuclear Magnetic Resonance spectra of cellulose (**a**) and cellulose-*g*-PNIPAAm (**b**) in different temperature conditions of 25 °C, 45 °C and 60 °C.

The obtained results showed that the variable-temperature ^1^H NMR experiments were used to confirm the thermo-responsive process of cellulose-*g*-PNIPAAm. Figure 8 showed a comparison of the MAS ^1^H NMR spectra of the cellulose and cellulose-*g*-PNIPAAm samples at 25 °C, 45 °C and 60 °C. The intensity of the characteristic chemical shift (3.9 ppm) in ^1^H NMR spectra of cellulose (Figure 8a) first increased and then decreased when the temperature was increased from 25 °C to 45 °C, then to 60 °C. This indicated that the hydrogen bonding between cellulose and water adsorbed on cellulose was disrupted at temperatures above 45 °C [46]. However, the intensity of the characteristic chemical shift (3.9 ppm) in ^1^H NMR spectra of cellulose-*g*-PNIPAAm (Figure 8b) increased when the temperature was increased from 25 °C to 60 °C, which is attributed to the enhancement and dominance of the intramolecular hydrogen bonding between the C=O and N–H groups in the PNIPAAm chains at temperatures above 45 °C [47].

**Figure 9:**
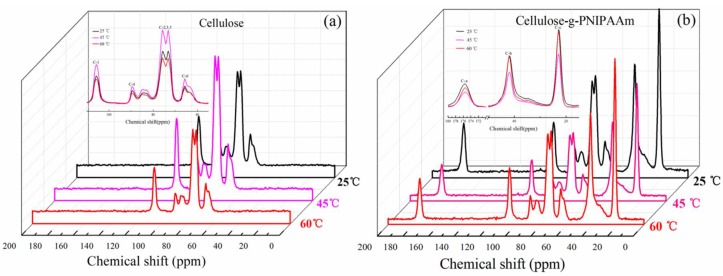
The in situ variable-temperature ^13^C solid-state Nuclear Magnetic Resonance spectra of cellulose (**a**) and cellulose-*g*-PNIPAAm (**b**) in different temperature conditions of 25 °C, 45 °C and 60 °C.

Furthermore, the in situ variable-temperature ^13^C NMR techniques were applied to monitor the thermo-responsive process of cellulose (Figure 9a) and cellulose-*g*-PNIPAAm (Figure 9b). The results showed that the intensity of the characteristic chemical shifts in the ^13^C NMR spectra of cellulose (Figure 9a) first increased and then decreased when the temperature was increased from 25 °C to 60 °C. Furthermore, the intensity of characteristic chemical shifts reached their maximum values when the temperature was 45 °C. This could be attributed to the mobility of cellulose molecules due to thermal motion, which enhanced the intermolecular hydrogen bonds of the cellulose molecules. Thus, the intensity of characteristic chemical shifts increased at 45 °C although it decreased after the temperature was 60 °C due to the intermolecular hydrogen bonds of the cellulose molecules being destroyed at a high temperature. In comparison with the cellulose, the intensity of the characteristic chemical shifts in the ^13^C NMR spectrum of cellulose-*g*-PNIPAAm (Figure 9b) first decreased and then increased when the temperature increased from 25 °C to 60 °C. The intensity of characteristic chemical shifts showed a turning point at 45 °C and reached their minimum values, indicating that the chemical environment of poly (*N*-isopropylacrylamide) on the surfaces of cellulose-*g*-PNIPAAm had been changed dramatically. This phenomenon could be due to the collapse of grafted PNIPAAm chains at 45 °C as the effort is greater than that of the expanding PNIPAAm chains caused by the thermal motion of molecule. However, when the temperature increased to 60 °C, the expanding PNIPAAm chains caused by the thermal motion of the molecules enhanced and dominated the grafted PNIPAAm chains itself, leading to the increase in the intensity of the characteristic chemical shifts [48].

On the basis of the earlier discussions, the thermo-responsive behaviour of PNIPAAm molecules of cellulose-*g*-PNIPAAm could be revealed as follows: at low temperatures, the PNIPAAm molecules on cellulose prefer to form hydrogen bonds with water molecules, leading to the hydrophilic state. With the increasing temperature, PNIPAAm molecules gradually form hydrogen bonds between the C=O and N–H groups in the PNIPAAm chains and become hydrophobic.

### 3.3. Controllable Oil/Water Adsorption Experiment

**Figure 10:**
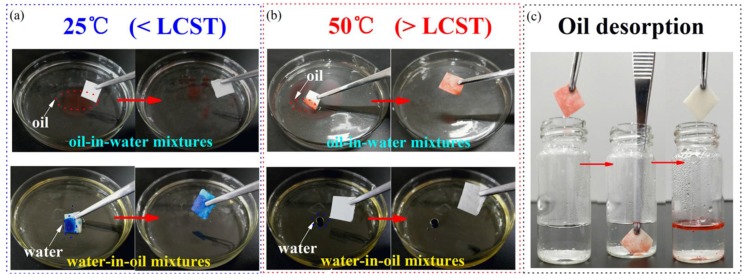
The adsorption behavior of cellulose-*g*-PNIPAAm paper in an oil/water mixture at 25 °C (**a**) and 50 °C (**b**), respectively. The inset of the top and bottom display the oil-in-water mixtures and water-in-oil mixtures, respectively. The paper pretreated with solutions of the oil layer (dyed red) on the water surface is shown in the top; the bottom shows the water droplet (dyed blue) under the oil; while there is depiction of the desorption behavior of the oil containing cellulose-*g*-PNIPAAm paper in 25 °C water (**c**).

As shown in Figure 10a, when the cellulose-*g*-PNIPAAm paper was immersed into the oil-in-water mixtures at 25 °C, it could not absorb the oil on the inside of the culture dish. However, it could absorb the water under the oil when immersed into the water-in-oil mixture. In addition, from Figure 10b, when the paper was immersed into the oil-in-water mixtures at 50 °C, it could absorb oil but it could not absorb the water under oil when immersed into the water-in-oil mixture. These phenomena indicate that the paper is hydrophilic and oleophobic at 25 °C, while it is hydrophobic and oleophilic at 50 °C. The results confirm the qualitative conclusion that the cellulose-*g*-PNIPAAm paper possesses a tunable wettability according to temperature adjustments. As a result, the maximum oil adsorption capacity of the soybean oil was about 9.74 g/g.

It is well known that oil-absorbing materials are easily fouled and can become clogged in the pore structures, making it difficult to reuse them. In this work, the thermo-responsive cellulose-*g*-PNIPAAm paper could not only separate the oil/water mixture, but also resist oil fouling. The cellulose-*g*-PNIPAAm paper could desorb the oil automatically in a water solution with a temperature of 25 °C after oil adsorption. In addition to the controllability of the surface wettability, its reversibility was also examined by recycling the temperature from 25 °C to 50 °C. After five cycles, there is only a negligible attenuation of the wettability, which indicates that the thermal-responsive switch of this modified surface is stable (Figure 10c).

### 3.4. Controllable Oil/Water Separation Experiment

**Figure 11:**
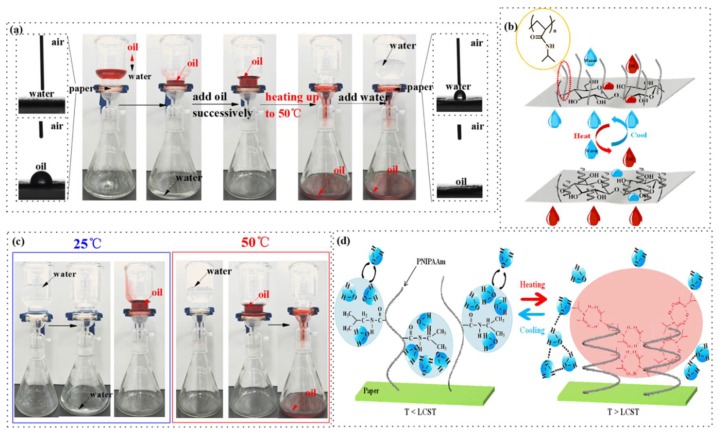
The time sequence of the oil/water separation process with cellulose-*g*-PNIPAAm paper (**a**). The oil/water mixture is composed of oil (dyed by sudan (III)/water). The left and right insets show the water droplet (**top**) and the oil droplet (**bottom**) on the corresponding cellulose-*g*-PNIPAAm, which is a schematic description (**b**); a contrast oil-only/water-only liquid filtration with the cellulose-*g*-PNIPAAm paper at 25 °C and 50 °C, respectively (**c**); the molecular mechanism diagram of the cellulose-*g*-PNIPAAm paper’s thermal-responsive wettability. Cellulose forms the main structure of the paper, while the reversible formation of the intermolecular and intra-molecular hydrogen bonding of PNIPAAm below and above the Lower Critical Solution Temperature exhibit the thermally-responsive wettability of the paper (**d**).

A series of proof-of-concept separation experiments was conducted to investigate the controllable oil/water separation capacity of cellulose-*g*-PNIPAAm paper via in situ temperature switching. The oil/water separation procedure was performed as shown in Figure 11a. The paper was fixed between two glass fixtures. Both of the fixtures were attached to a glass tube and placed vertically.

When the oil/water mixture was poured, the water passed through the paper rapidly, while the oil was repelled and kept in the upper glass tube because of the excellent hydrophilic and oleophobic properties. During the separation, no external forces were used except gravity (Figure 11a). After the separation, the water was collected in the beaker. Meanwhile, the paper was in situ heated with an 800 W hair dryer for 2.5 min. When the PNIPAAm reached its LCST (45 °C) or above, the paper’s water resistance capacity was significantly increased so that the oil passed through the paper easily, while water was repelled and kept in the upper glass tube because of the excellent hydrophobic and oleophilic properties. Additionally, the water flux F was calculated here, where *V* was fixed to 50 mL, *S* was the effective area of the paper surface (4π cm^2^) and *t* was the required time (15 s) for the permeation of 50 mL of water. As a result, the water flux was calculated to be 2.65 ± 0.36 L m^−2^ s^−1^, indicating that the as-prepared paper is capable of separating a large amount of oil/water mixtures.

To further investigate the dynamic transition of wetting behaviors on the surface of the cellulose-*g*-PNIPAAm paper, water contact angles (WCA) and oil contact angles (OCA) were measured in the surface of the paper under different temperature conditions. Figure 10b displays the WCA and OCA of the paper at 25 °C and 45 °C, respectively. When the temperature was 25 °C, the WCA of the paper was about 0°, while the OCA was about 72.0 ± 0.6°. When the temperature was raised to 45 °C, the WCA of the paper was about 134.2 ± 0.6°, while the OCA was about 9.1 ± 0.4°. This can contribute to the enhanced hydrophobic interactions of the isopropyl group of PNIPAAm and a significant decrease in hydrogen bonding between the water molecules and grafted PNIPAAm. Due to the superhydrophobicity and oleophilic properties, the cellulose paper cannot selectively separate oil from the oil/water mixtures with a WCA of 0° and an OCA of 68.4 ± 0.8° at 25 °C,. Furthermore, it does not have temperature-sensitive performance, indicating that it has no potential for oil/water separation (see details in supporting information).

## 4. Conclusions

A thermo-responsive cellulose-*g*-PNIPAAm material was successfully prepared by grafting *N*-isopropylacrylamide (NIPAAm) onto bagasse pulp cellulose via Ce (IV)-initiated free radical polymerization. The modified cellulose material showed a good reversible switching from being hydrophilic to becoming hydrophobic in response to changes in temperature. The as-prepared paper realized controllable oil/water separation with an efficiency separation flux of 2.65 ± 0.36 L m^−2^ s^−1^. It can selectively separate water from oil/water mixtures and it can allow water and oil to permeate through the paper in order to be collected separately at temperatures above 45 °C. Meanwhile, the separation efficiency invariably remained even after five cycles’ application, indicating a promising regeneration performance. More importantly, the thermo-responsive mechanism was characterized by in situ variable-temperature NMR and AFM analysis. The results demonstrated that the competition of inter-molecular/intra-molecular H-bonding between the C=O and N–H groups in the PNIPAAm chains in response to temperature leads to a good reversible switching from being hydrophilic to becoming hydrophobic within a narrow range.

## Supplementary Materials

The following are available online at http://www.mdpi.com/2073-4360/10/6/592/s1, Figure S1: Scanning Electron Microscopy and Energy Dispersive Spectroscopy of the cellulose (a) and cellulose-*g*-PNIPAAm (b), Figure S2: The time sequence of the oil/water separation process with the cellulose paper. The oil/water mixture is composed of oil dyed by Sudan (III)/water. The left and right inset shows the water droplet (top) and oil droplet (bottom) on corresponding cellulose. Table S1: The element content of cellulose and cellulose-*g*-PNIPAAm measured by Elemental Analysis. Table S2: The element content of cellulose and cellulose-*g*-PNIPAAm measured by X-ray Photoelectron Spectroscopy.
