# Supplementary material for: Thermo-Responsive Cellulose-Based Material with Switchable Wettability for Controllable Oil/Water Separation
Source: Polymers (Basel). 2018 May 28;10(6):592. doi: 10.3390/polym10060592 (PMC6403979; doi:10.3390/polym10060592)
Supplement: Supplementary file 1 [file polymers-10-00592-s001.zip › polymers-301779-SI/polymers-301779-SI.pdf]

polymers MDPI

Supplementary Materials

Thermo-Responsive Cellulose-Based Material with Switchable Wettability for Controllable Oil/Water Separation

Wenbo Chen [1,2], Hui He [1,2,*], Hongxiang Zhu [1,2,*], Meixiao Cheng [1,2], Yunhua Li [1,2] and Shuangfei Wang [1]

[1] College of Light Industry and Food Engineering, Guangxi University, Nanning 530004, China; ilaureshang@163.com (W.C.); cmx29192@163.com (M.C.); liyunhua199202@163.com (Y.L.); wangsf@gxu.edu.cn (S.W.)
[2] Guangxi Key Laboratory of Clean Pulp & Papermaking and Pollution Control, Nanning 530004, China
* Correspondence: gxuhh@gxu.edu.cn (H.H.); zhx@gxu.edu.cn (H.Z.);
Tel.: +86-18260864618 (H.H.); +86-13978154707 (H.Z.)

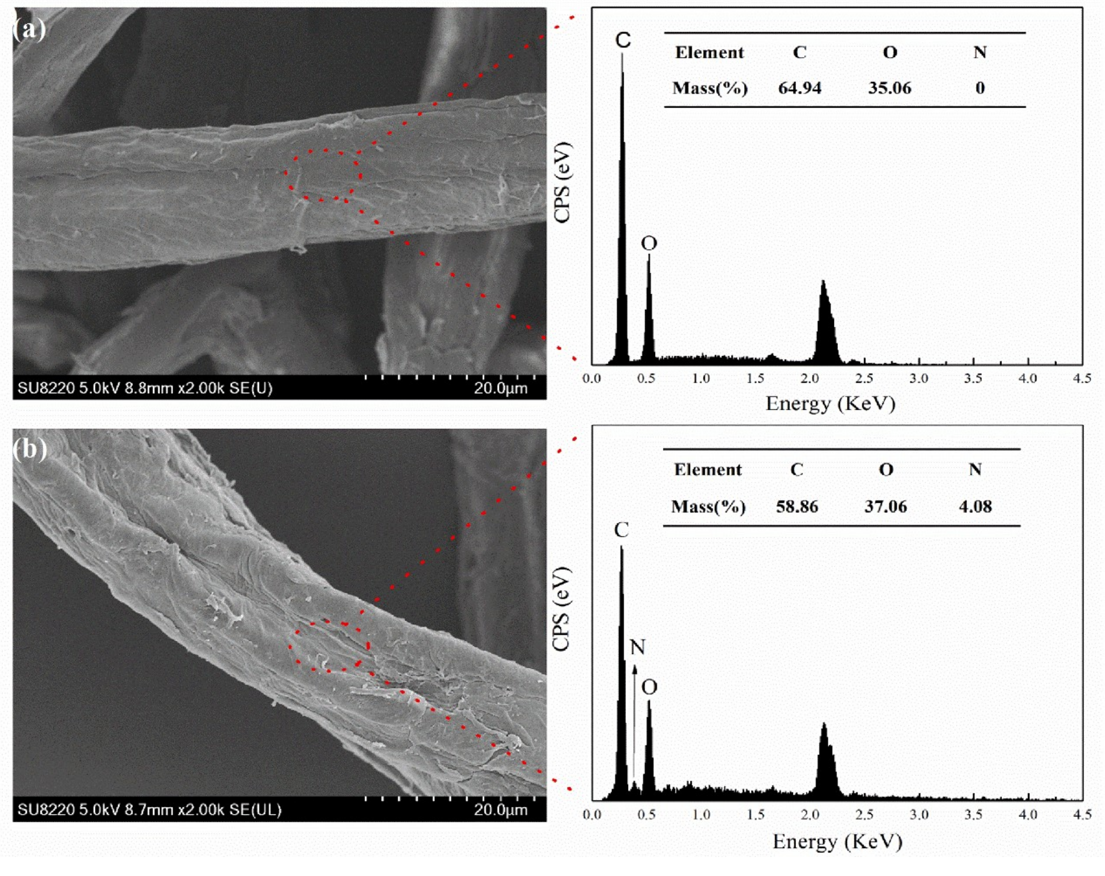

Figure S1. Scanning Electron Microscopy and Energy Dispersive Spectroscopy of the cellulose **(a)** and cellulose-g-PNIPAAm **(b)**

The surface micromorphology and element composition of the cellulose and cellulose-g-NIPAAm were shown in Figure S1 and Table S1. In Figure S1, it was evident that cellulose and cellulose-g-PNIPAAm have fibrous structures and that the inhomogeneous polymers were covered on the surface of cellulose-g-PNIPAAm; combinating from the analyses of EA (Table S1), XPS (Table S2), and FT-IR (Figure 2), the inhomogeneous polymers should be PNIPAAm.

Elemental analysis and XPS were employed to investigate the grafting degree of PNIPAAm on cellulose. As shown by the EA results (Table S1), no nitrogen was detected in the cellulose, but the total nitrogen content of cellulose-g-PNIPAAm increased with the graft degree (G, %).

Table S1. The element content of cellulose and cellulose-g-PNIPAAm measured by Elemental Analysis.

**Sample**	**EA**			**G_{EA} (%)**
	**C (%)**	**H (%)**	**N (%)**	
**Cellulose**	41.21	6.42	0	---
**Cellulose-g-PNIPAAm 1**	44.41	6.95	1.48	13.51
**Cellulose-g-PNIPAAm 2**	44.56	6.77	1.77	16.55
**Cellulose-g-PNIPAAm 3**	47.08	7.72	3.71	42.28
**Cellulose-g-PNIPAAm 4**	45.24	7.13	4.24	51.45
**Cellulose-g-PNIPAAm 5**	48.66	7.45	4.36	53.68

Table S2. The element content of cellulose and cellulose-g-PNIPAAm measured by X-ray Photoelectron Spectroscopy

**Sample**	**XPS**			**G_{XPS} (%)**
	**C (%)**	**O (%)**	**N (%)**	
**Cellulose**	67.99	32.01	0	---
**Cellulose-g-PNIPAAm 1**	73.85	24.16	1.99	18.97
**Cellulose-g-PNIPAAm 3**	68.35	26.16	5.50	78.76

EA tests the elemental content of the bulk of material, indeed, XPS has been employed to analyze the surface chemical properties of cellulose-g-PNIPAAm (Figure 4 in the manuscript). in the 10 nm range. In Table S1 and Table S2, the whole and the surface nitrogen element content of cellulose-g-PNIPAAm was 3.71 % and 5.50 % by EA and XPS, respectively. Nitrogen content of 5.50 % according to the data obtained from wide-scan spectra of cellulose-g-PNIPAAm shows that the graft percent of PNIPAAm on cellulose was about 78.76 % which is further the value resulted from elemental analysis. It could be attributed to the introducing of PNIPAAm onto the surface of the cellulose. For cellulose, the O/C value was calculated as 0.47 and for cellulose-g-PNIPPAm it was 0.33 and 0.38. As

expected, the O/C values for the modified cellulose followed decreased trend because of the higher corbon content in the PNIPAAm, which was the further proof for the surface modification of cellulose.

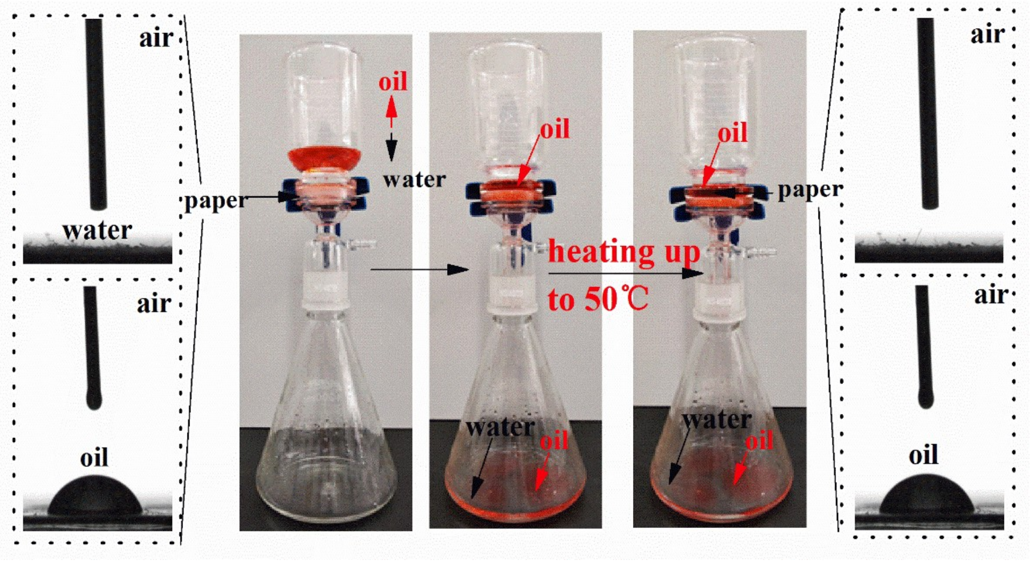

Figure S2. The time sequence of the oil/water separation process with the cellulose paper. The oil/water mixture is composed of oil dyed by Sudan (III)/water. The left and right inset shows the water droplet (top) and oil droplet (bottom) on corresponding cellulose

A series of Separation experiments have been carried out to test the controllable oil/water separation capacity on cellulose paper via in situ temperature switching separately. The oil/water separation procedure was performed as showed in Figure S2.

When the mixture of oil and water was poured, water can immediately through the paper and trapped quickly in the rough structure via capillary force, the floating oil shrinks slowly and cannot through the paper completely after a while, in this case WCA was about 0°, OCA was about 68.4 ± 0.8°. Followed the paper was in situ heated with an 800 W hair dryer for 2.5 min, when the temperature was increased to 50 °C, the result was absolutely the same with the WCA of 0° and the OCA of 66.2 ± 0.5°. As a result, cellulose paper showed hydrophilic and oleophylic property in the water/solid two-system, it also do not have the temperature sensitive response performance, thus has no potential for oil/water separation.
