# Supplementary material for: Thermo-Responsive Cellulose-Based Material with Switchable Wettability for Controllable Oil/Water Separation
Source: Polymers (Basel). 2018 May 28;10(6):592. doi: 10.3390/polym10060592 (PMC6403979; doi:10.3390/polym10060592)
Supplement: Supplementary file 1 [file polymers-10-00592-s001.zip › polymers-301779-SI/polymers-301779-SUPPL-HIGHLIGHT.pdf]

Highlights

- A novel thermo-responsive cellulose-based material for oil/water separation was prepared and characterized.
- The material could selectively separate oil from the oil/water mixture by adjusting the temperature.
- The material exhibited a good regeneration performance.
- A possible thermo-responsive mechanism was recovered by In-situ variable-temperature NMR and AFM analysis.
